# Effects of incubation temperature and *in ovo* supplementation of glucose and vitamin D_3_ on eggshell temperature, embryonic mortality, hatchability, chick quality, and post-hatch performance in broiler chickens

**DOI:** 10.3389/fphys.2026.1930808

**Published:** 2026-09-23

**Authors:** Maxwell Ansong Okai, Francis Kruenti, Yendouchamtchié Nadiedjoa, Clarice Temhoul Maa Maa, Ahmed Mijiyawa, Lijing Sun, Jumeng Yuan, Minghui Wang, Kokou Tona, Jacob Alhassan Hamidu, Benjamin Adjei-Mensah, Felix Kwame Amevor, Hai Lin

**Affiliations:** 1 Key Laboratory of Efficient Utilization of Non-grain Feed Resources (Co-construction by Ministry and Province), Ministry of Agriculture and Rural Affairs, Shandong Provincial Key Laboratory of Animal Nutrition and Efficient Feeding, Department of Animal Science, Shandong Agricultural University, Taian, Shandong, China; 2 Council for Scientific and Industrial Research, Animal Research Institute, Achimota, Accra, Ghana; 3 Regional Center of Excellence on Sciences at the University of Lomé, Lomé, Togo; 4 Department of Animal Science, Faculty of Agriculture, Kwame Nkrumah University of Science and Technology, Kumasi, Ghana; 5 Department of Animal Science, University of Ghana, Legon, Ghana

**Keywords:** eggshell temperature, glucose, *in ovo* feeding, incubation temperature, post-hatch performance, vitamin D3

## Abstract

**Introduction:**

Incubation temperature and embryonic nutrient availability are critical determinants of successful embryogenesis and early post-hatch performance in broilers. *In ovo* feeding has emerged as an effective strategy to enhance embryonic nutrient supply. However, the efficiency of nutrient utilization by developing embryos may be dependent on the incubation conditions. Therefore, the present study investigated the interactive effects of incubation temperature and *in ovo* supplementation of glucose or vitamin D_3_ on eggshell temperature, hatchability, chick quality, and post-hatch growth performance of broilers.

**Methods:**

A total of 1,200 fertile eggs obtained from 58-week-old Arbor Acres breeder hens (62 ± 1 g) were incubated in an automatic dual-system incubator (Senjie DZ 47-53) at three machine air temperatures (36.5°C, 37.0°C, or 37.5°C), with relative humidity maintained at 60%. The assigned thermal treatments commenced on embryonic day (ED) 1 and were maintained continuously through ED18, with eggs remaining at their respective incubation temperatures (36.5°C, 37.0°C, or 37.5°C) throughout this incubation period. Each temperature treatment consisted of 400 eggs with four replicates. After candling on ED10, eggs within each incubation temperature treatment were randomly allocated to one of three *in ovo* treatments: a control group (eggs that were perforated but uninjected), a glucose injection group (5 mg/ml), or a vitamin D_3_ injection group (25 mg/ml). Thus, each incubation temperature group was subdivided into the three *in ovo* treatment groups on ED10, allowing the effects of incubation temperature, *in ovo* treatment, and their interaction to be evaluated. Following completion of the thermal treatment on ED18, all eggs were transferred to the hatcher for completion of embryonic development and hatching. The eggshell temperature was monitored throughout the incubation period, and the embryonic mortality, hatchability, chick quality traits, and post-hatch growth performance were evaluated.

**Results:**

The results showed that the incubation temperature significantly influenced the eggshell temperature, with higher values recorded at 37.0°C and lower values at 36.5°C (*p* < 0.05). However, *in ovo* feeding also affected the eggshell temperature, which was generally lower in glucose-treated eggs and highest in vitamin D_3_-treated eggs (*p* < 0.05). Hatchability increased with increasing incubation temperature, reaching the highest value at 37.5°C (81.92%). The greatest hatchability (89.86%) was observed in glucose-injected eggs incubated at 37.5°C. In addition, embryonic mortality was significantly higher in the control group, whereas the glucose group exhibited the lowest mortality compared with the vitamin D_3_ and control groups (*p* < 0.05). Furthermore, incubation at 37.5 C significantly reduced embryonic mortality compared with the 36.5 C and 37.0 C treatments (*p* < 0.05). The chick hatching weight was highest in the vitamin D_3_-treated eggs incubated at 37.5 C (51.72 g). The incubation temperature × *in ovo* feeding interaction significantly improved feed intake, with the highest intake recorded in birds from the glucose-injected eggs incubated at 37.0°C, proceeded by birds from the vitamin D_3_-injected eggs at the same temperature. Furthermore, for the post-hatch growth traits, broilers originating from eggs incubated at 37.0°C exhibited the greatest final body weight (1,283.52 g) and body weight gain (1,234.80 g; *p* < 0.05) at 42 days of age.

**Conclusion:**

Taken together, these findings demonstrate that the incubation temperature significantly influenced eggshell temperature, whereas *in ovo* glucose and vitamin D_3_ administration at 37.5°C improved hatchability, whereas incubation at 37.0°C supported superior post-hatch growth performance. The combined optimization of incubation temperature and embryonic nutrient supplementation may therefore enhance the embryonic response, hatchery efficiency, and broiler production outcomes.

## Introduction

Incubation conditions play a pivotal role in determining the embryonic development, hatchability, and subsequent performance of broiler chickens (Yalcin et al., 2022). Among these factors, temperature is considered as the most critical environmental determinant of embryonic metabolism and development during incubation (Bednarczyk et al., 2016; Decuypere et al., 2001). Even slight deviations from the optimal incubation temperature can alter the embryonic growth rate, organ development, and chick quality at hatch, thereby affecting post-hatch performance (Decuypere et al., 2001). In addition to the incubation conditions, the availability of nutrients to the developing embryo is another key factor influencing embryogenesis and early chick viability (Tona et al., 2022; Yalcin et al., 2022). In recent years, *in ovo* feeding has received considerable attention as a nutritional strategy aimed at enhancing the nutrient supply available to the developing embryo. This technique involves the direct administration of nutrients into the egg during incubation to support embryonic growth and improve chick quality at hatch. Previous studies have shown that early nutrient supplementation through *in ovo* feeding can influence gene expression, enhance organ development, and improve early physiological functions in poultry embryos (Bednarczyk et al., 2016). Therefore, *in ovo* feeding has been increasingly explored as a method to improve hatchability and post-hatch performance in broiler production systems.

The environmental conditions during incubation may also interact with embryonic nutrition. In poultry science, the influence of incubation environment on embryonic development and chick quality has been extensively investigated. Studies have demonstrated that both incubation temperature and early *in ovo* nutrient supplementation can significantly affect the hatchability characteristics and overall chick health (Zhang et al., 2018; Xu et al., 2023). *In ovo* supplementation is designed to enhance the utilization of nutrients within fertile eggs and thereby support embryogenesis, hatchability, and post-hatch growth. However, the metabolic utilization of these nutrients by the developing embryo may be influenced by the incubation temperature, which regulates the embryonic metabolic rate and physiological activity (Foye et al., 2006; Zhai et al., 2011).

Among the various nutrients investigated for *in ovo* supplementation, glucose and vitamin D_3_ are of particular interest due to their important physiological roles during embryonic development. Glucose serves as a readily available energy source that supports the high metabolic demands of the developing embryo, especially during the later stages of incubation when the energy requirements increase (Moran, 2007). However, vitamin D_3_ plays a fundamental role in calcium and phosphorus metabolism, skeletal formation, immune regulation, and overall embryonic development (Di Rosa et al., 2011; Hrabia et al., 2023). Despite their recognized biological importance, there is limited information available regarding how the effectiveness of these nutrients may be influenced by incubation temperature conditions.

Incubation temperature and embryonic nutrient availability are closely interconnected determinants of embryonic development. Temperature regulates the embryonic metabolic rate, oxygen demand, tissue development, and the timing of developmental transitions, whereas *in ovo* nutrient supplementation directly modifies the availability of substrates or micronutrients to the developing embryo (Agyekum et al., 2022). However, the physiological response to an *in ovo* nutrient may be dependent on the thermal environment in which embryonic metabolism occurs. A temperature that alters metabolic activity may change the rate at which an injected nutrient is utilized, while additional nutrient availability may, in turn, influence embryonic metabolic activity and heat production. Eggshell temperature provides a noninvasive indicator of the thermal relationship between the embryo and its surrounding environment and can therefore provide additional information beyond the programmed machine temperature (Kpodo and Proszkowiec-Weglarz, 2023). This interaction is particularly relevant during the middle and late stages of incubation, when the embryonic metabolic activity increases and the embryo becomes progressively more dependent on efficient nutrient mobilization and utilization (Tona et al., 2022). Glucose can provide an immediately available energy substrate, whereas vitamin D_3_ is involved in calcium and phosphorus metabolism and skeletal development (Girgis et al., 2015; Okai et al., 2026). Therefore, the physiological consequences of these supplements may not be uniform across incubation temperatures. Examining the incubation temperature and *in ovo* supplementation simultaneously may consequently provide a more informative assessment of embryonic responses than evaluating either factor independently. Based on this, the present study evaluated whether the effects of glucose or vitamin D_3_ supplementation on eggshell temperature, embryonic survival, hatchability, chick quality, and subsequent growth performance differed according to the incubation temperature.

## Materials and methods

### Ethical approval

All experimental procedures were approved by the Animal Care and Use Committee of Shandong Agricultural University (Taian, China) and were conducted in accordance with the Guidelines for Experimental Animals issued by the Ministry of Science and Technology of the People’s Republic of China.

### Preparation and sterilization of *in ovo* feed supplements

All equipment used for the preparation and administration of the *in ovo* feed supplements was sterilized in a Panasonic MLS-3781 autoclave and subsequently dried in an Electric Blast Drying Oven (Shanghai-Heng Scientific Instrument Co. Ltd., Shanghai, China) prior to use. The glucose solution was prepared by dissolving glucose in phosphate-buffered saline (PBS) to obtain a concentration of 5 mg/ml. For the vitamin D_3_ solution, vitamin D_3_ was first dissolved in anhydrous ethanol to enhance solubility, after which PBS was added to obtain a final concentration of 25 mg/ml. The glucose and vitamin D_3_ concentrations were selected based on previously evaluated *in ovo* administration protocols using the same route and comparable experimental conditions. Glucose was prepared at 5 mg/ml and vitamin D_3_ at 25 mg/ml, and 0.2 ml of each respective solution was administered per egg. Thus, on incubation day 10, each egg was injected with 0.2 ml of the respective supplement through the shell overlying the air sac at the blunt end of the egg using an automatic syringe at egg level, as described by Kuka et al. (2023). The injection was performed on a UV-sterilized table. A small perforation was made through the eggshell over the air sac to facilitate insertion of the syringe, and the injection site was immediately sealed with wax after administration. The eggs were then returned to their respective incubators. These concentrations were selected as predefined experimental doses rather than as putative optimal doses. The present experiment was designed to investigate the interaction between incubation temperature and embryonic supplementation; therefore, dose optimization or dose–response relationships are beyond the scope of the present study. Furthermore, the fertilized eggs were incubated in an automatic dual-system incubator (Senjie DZ 47-53) at three machine air temperatures—36.5°C, 37.0°C, or 37.5°C—with relative humidity maintained at 60% from embryonic day (ED) 1 to ED18.

On ED10, eggs assigned to the treatment groups were injected with 0.2 ml of the respective solutions through the air sac using 1-ml sterile disposable syringes. ED10 was selected based on previous studies demonstrating effective embryonic nutrient uptake during mid-incubation while minimizing embryo injury risk (Dayan et al., 2023; Okai. et al., 2025a). All injections were performed on a UV-sterilized work surface that had been disinfected with sterile saline and anhydrous alcohol to maintain aseptic conditions. After injection, the perforation sites were sealed with sterile wax, and the eggs were returned to their respective incubators for further incubation.

### Experimental design

A total of 1,200 fertile broiler breeder eggs (62 ± 1 g) were obtained from a 58-week-old Arbor Acres breeder flock. The eggs were randomly allocated to three incubation temperature treatments—36.5°C, 37.0°C, and 37.5°C—with 400 eggs assigned to each temperature treatment. The incubation temperatures were selected to represent a conventional reference condition and experimentally controlled lower-temperature conditions. The 37.5°C treatment represented the conventional machine air incubation temperature used as the reference condition, whereas 37.0°C and 36.5°C were included to generate a controlled thermal gradient for evaluating temperature-dependent responses to *in ovo* supplementation. The purpose of including these lower temperatures was not to recommend them for commercial hatchery use but to determine whether the effects of glucose and vitamin D_3_ supplementation were modified by the thermal environment. Previous studies have demonstrated that the broiler embryonic development, eggshell temperature, hatching characteristics, and post-hatch performance can respond to experimentally imposed differences in incubation temperature (Hamidu et al., 2018; Agyekum et al., 2022; Yalcin et al., 2022). Thus, the three temperature levels enabled the temperature°×°*in ovo* supplementation interaction to be evaluated within a controlled factorial design. Within each incubation temperature, the eggs were distributed among four independent replicate groups, with each replicate maintained in a separate automatic dual-system incubator (Senjie series DZ47-53, Shanghai, China; capacity, 528 eggs). Relative humidity was maintained at 60%, and the assigned incubation temperature was maintained from ED1 to ED21. On ED18, the eggs were transferred to the hatching compartment of the same incubator, which was maintained at the same temperature (36.5°C, 37.0°C, and 37.5°C) until hatch.

On ED10, following candling, the eggs within each incubation temperature treatment were randomly assigned to one of three *in ovo* feeding treatments: control (eggs that were perforated and handled similarly, but were not injected), glucose (eggs that received a glucose solution), or vitamin D_3_ (eggs that received a vitamin D_3_ solution). Thus, the experiment followed a 3 × 3 factorial arrangement consisting of three incubation temperatures and three *in ovo* feeding treatments. Each of the nine temperatures × *in ovo* feeding combinations contained four independent replicates, resulting in a total of 36 experimental units.

The replicate, rather than the individual egg, was considered the experimental unit because eggs within a replicate shared the same incubation and treatment conditions and therefore could not be considered statistically independent observations. Accordingly, measurements obtained from individual eggs or chicks within the same replicate were summarized as a replicate-level mean before statistical analysis.

Eggshell temperature was monitored from ED1 to ED20. The eggs used for eggshell temperature measurements were distributed among the corresponding replicate groups. For each incubation day, eggshell temperature measurements obtained from eggs belonging to the same replicate were averaged, and the resulting replicate mean was used as the statistical observation.

At hatch, 10 chicks were randomly selected from each treatment group for assessment of the chick quality traits, including body weight, chick length, and shank length. Because multiple chicks originated from the same experimental replicate, measurements obtained from chicks belonging to the same replicate were averaged, and the replicate mean was used for statistical analysis. Thus, individual eggs and chicks were not treated as independent experimental observations.

This experimental structure ensured that the four replicates within each temperature × *in ovo* feeding combination represented four independent experimental units for statistical analysis.

### Animal management

The chicks were housed in battery cages measuring 38 cm in width (*W*) × 47 cm in length (*L*) × 35 cm in height (*H*), with 10 birds per cage. The chicks were maintained under the same management conditions throughout the post-hatch experimental period. At hatch, 10 chicks were randomly selected from each of the nine treatment combinations (three incubation temperatures × three *in ovo* treatments), resulting in a total of 90 chicks for the assessment of chick quality traits, including body weight, chick length, and shank length. The experimental facility was maintained at a temperature of 23 ± 2°C and a relative humidity of 65% ± 3% throughout the study period. The birds were provided *ad libitum* access to a commercial starter and finisher diet according to the nutrient requirements recommended by the National Research Council (NRC, 1994), containing 23% crude protein and 3,000 kcal/kg metabolizable energy. Fresh drinking water was supplied *ad libitum* through nipple drinkers. The Gumboro and first Newcastle vaccines were administered to the birds on days 7 and 14, respectively, but no medications were provided.

#### Measurement of eggshell temperature

With a human infrared camera (TP160 Serno: 900075013198), the eggshell temperature was measured at the equator of 80 randomly selected eggs from each subgroup at eight eggs each from the upper, middle, and lower positions of each horizontal rack in the incubators. Furthermore, the eggshell temperature of each egg was measured at 6:00 a.m., 12:00 p.m., and 6:00 p.m (Agyekum et al., 2022).

#### Hatching events (external pipping and hatching times)

The external pipping and hatching times were measured from the 19th day of incubation by examining the remaining eggs at each of the three incubation temperatures at intervals of 6 h (6 a.m., 12 p.m., 6 p.m., and 12 a.m.). After each check, the eggs or chicks were marked to help distinguish them from others that would pip or hatch at the subsequent check 6 h later. This helped to prevent confusion regarding which chicks were pipping or hatching.

#### Measurement of embryonic mortality, hatchability, and chick quality parameters

Embryonic mortality was calculated as the percentage of fertile eggs in which the embryo dies during incubation before hatching. The hatchability of fertile eggs was calculated as the ratio of chicks hatched to the number of fertile eggs, expressed as a percentage. A total of 90 chicks from each treatment group were randomly evaluated for their body weight, body length, and shank length. The chick hatching weight was measured immediately after the chicks were pulled from the hatcher with an Ohaus External Chemical Balance (SP602AM; 165 mm × 142 mm, 2,000 g; resolution, 0.1 g). A pair of dividers and a ruler were used to measure chick length, i.e., the distance between the tip of the beak and the end of the middle toe, and shank length, i.e., the distance from the tibiotarsal–tarsometatarsal joint to the pad between the third and fourth toes (Agyekum et al., 2022).

#### Measurement of post-hatch growth traits

At 42 days of age, the individual body weight of chicks in each cage was taken with the Ohaus External Chemical Balance. The feed intake and body weight gain (BWG) were recorded weekly and were used to calculate the feed conversion ratio (FCR) using the following formula:


Feed intake=Total feed supplied–Total feed left

Body weight gain=Final body weight–iInitial body weight

$$\text{FCR}=\frac{\text{Total feed Iintake}}{\text{Total body weight gain }}$$

### Statistical analysis

The experiment was analyzed as a 3 × 3 factorial design using a two-way analysis of variance (ANOVA), with incubation temperature and *in ovo* feeding treatment as the two fixed factors. Incubation temperature included three levels (36.5°C, 37.0°C, and 37.5°C), and the *in ovo* feeding treatment included three levels (control, glucose, and vitamin D_3_). The replicate was considered as the experimental unit. Therefore, individual eggs or chicks within a replicate were not considered independent observations. All measurements obtained from eggs or chicks within the same replicate were first averaged to obtain a single replicate-level observation before statistical analysis. Thus, each temperature × *in ovo* feeding combination contributed four independent replicate observations, resulting in 36 experimental observations for the factorial analysis.

The statistical model was:


$${\text{Y}}_{\text{i}\text{j}\text{k}}=\text{μ}+{\text{TEMP}}_{\text{i}}+{\text{IOF}}_{\text{j}}+{\left(\text{TEMP}\times \text{IOF}\right)}_{\text{i}\text{j}}+{\text{e}}_{\text{i}\text{j}\text{k}}$$

where *Y_ijk_* is the response variable for replicate *k* receiving incubation temperature *i* and *in ovo* feeding treatment *j*; *μ* is the overall mean; TEMP*_i_* is the fixed effect of incubation temperature; IOF*_j_* is the fixed effect of *in ovo* feeding treatment; (TEMP × IOF)*_ij_* is the interaction between incubation temperature and *in ovo* feeding treatment; and *eij_k_* is the random residual error associated with replicate *k* within the corresponding temperature × *in ovo* feeding treatment combination.

The analysis was performed using the General Linear Model procedure in Minitab version 19. When significant main effects or interactions were detected (*p* < 0.05), the treatment means were separated using Tukey’s honestly significant difference (HSD) test.

## Results

### Effects of eggshell temperature pre *in ovo* feeding

Prior to *in ovo* feeding, the eggshell temperature was significantly influenced by the incubation temperature (*p* < 0.05). As shown in **Table 1**, the eggshell temperature increased progressively as the incubation temperature increased from 36.5°C to 37.0°C and 37.5°C during the early incubation period (ED1–ED9). During the early incubation period (ED1–ED9), the eggshell temperature generally reflected the programmed incubation temperature, although day-to-day fluctuations were observed. As shown in **Table 1**, incubation period, although the overall pattern remained associated with the machine-set incubation temperatures.

**Table 1:** Effects of incubation temperature on eggshell temperature before *in ovo* feeding.

Incubation day	Temperature (°C)			SEM	*p*-value
	36.5	37.0	37.5		
ED1	33.88c	34.72b	36.03a	0.0907	<0.0001
ED2	34.56c	35.97b	36.83a	0.0969	<0.0001
ED3	34.57c	36.58b	37.86a	0.0613	<0.0001
ED4	35.48c	36.84b	37.48a	0.0830	<0.0001
ED5	35.55c	36.41b	37.36a	0.0763	<0.0001
ED6	36.06c	36.68b	37.38a	0.0692	<0.0001
ED7	35.74c	36.87b	37.55a	0.0636	<0.0001
ED8	36.23c	36.73b	37.33a	0.0678	<0.0001
ED9	35.59c	36.50b	36.98a	0.0742	<0.0001
ED10	35.71b	36.04b	36.70a	0.113	<0.0001

Means with different lowercase letters in a row are significantly different. Probability value (*p* < 0.05).

*ED*, experimental/incubation day; *SEM*, standard error of the mean.

### Effects of eggshell temperature post *in ovo* feeding

After *in ovo* feeding, the eggshell temperature was significantly affected by treatment during several stages of incubation. As presented in **Table 2**, *in ovo* feeding significantly influenced the eggshell temperature on incubation days 12, 14, 17, 18, 19, and 20 (*p* < 0.05). A significant interaction between incubation temperature and *in ovo* feeding was also observed across the incubation period. At 36.5°C, the eggshell temperature was generally lowest in the control group, intermediate in the glucose-injected eggs, and highest in the vitamin D_3_-injected eggs. At 37.0°C, eggs that received vitamin D_3_ injection exhibited the lowest eggshell temperature, followed by the glucose-injected eggs, whereas the control group recorded the highest eggshell temperature values.

**Table 2:** Effects of incubation temperature on eggshell temperature after *in ovo* feeding.

Factor	Feeding group	Incubation day									
		ED11	ED12	ED13	ED14	ED15	ED16	ED17	ED18	ED19	ED20
*In ovo* feeding	CON	36.52	36.63a	36.73	36.64ab	36.33	36.74	36.75ab	36.39	35.78b	35.58ab
	GLU	36.30	36.11b	36.70	36.48b	36.34	36.57	36.57b	36.57	35.10c	35.11b
	VIT	36.48	36.31ab	36.69	37.84a	36.34	36.69	36.84a	36.43	36.32a	35.79a
	SEM	0.0814	0.109	0.0623	0.0643	0.0584	0.0691	0.0679	0.0720	1.33	**0.129**
Incubation temperature (°C)	36.5	35.31c	35.03c	36.00c	36.59b	35.53c	35.52c	35.50b	35.46c	35.24b	35.54
	37.0	37.31a	37.28a	36.88b	37.23a	36.42b	37.10b	37.42a	36.66b	36.38a	35.64
	37.5	36.86b	36.74b	37.24a	37.14a	37.08a	37.37a	37.30c	37.27a	35.38b	35.30
	SEM	0.0814	0.109	0.0623	0.0643	0.0584	0.0691	0.0679	0.0720	1.33	**0.129**
*In ovo* feeding × incubation temperature (°C)	CON*36.5	34.76e	34.62c	35.76d	35.65c	35.38e	35.38c	35.44c	35.17d	34.78bc	35.10c
	CON*37.0	37.83a	38.02a	37.20ab	37.23ab	36.57cd	37.46a	37.50a	36.87b	36.79a	36.32ab
	CON*37.5	36.96bc	37.26ab	37.23ab	37.05ab	37.05ab	37.38ab	37.28ab	37.15ab	35.16bc	35.32bc
	GLU*36.5	35.43d	35.02c	36.14d	35.45c	35.82e	35.71c	35.53c	35.64d	34.33c	35.09c
	GLU*37.0	37.06b	36.88b	36.79bc	37.15ab	36.41cd	36.85b	37.40a	36.89b	35.38b	34.90c
	GLU*37.5	36.41c	36.43b	37.18ab	36.85b	36.85bc	37.24ab	36.79b	37.19ab	35.62b	35.33bc
	VIT*36.5	35.76d	35.44c	36.12d	35.68c	35.39e	35.71c	35.44c	35.59d	36.60a	36.44a
	VIT*37.0	37.04bc	36.96b	36.63c	37.31ab	36.28d	36.85b	37.33a	36.22c	36.98a	35.70abc
	VIT*37.5 SEM	36.70bc 0.141	36.53b 0.189	37.31a 0.108	37.51a 0.111	37.35a 0.101	37.51a 0.120	37.76a 0.118	37.48a 0.125	35.37b 1.78	35.24c **0.223**
*p*-value	IOF	0.130	0.005	0.902	0.001	0.949	0.204	0.022	0.192	<0.0001	**0.001**
	Temp	<0.0001	<0.0001	<0.0001	<0.0001	<0.0001	<0.0001	<0.0001	<0.0001	<0.0001	**0.159**
	IOF*Temp	<0.0001	<0.0001	<0.0001	0.045	<0.0001	0.003	<0.0001	<0.0001	<0.0001	**<0.0001**

Means with different lowercase letters in a column are significantly different.

ED, experimental/incubation day; CON, control; GLU, glucose; VIT, vitamin; IOF, in ovo feeding; Temp, temperature; SEM, standard error of the mean.

Bold formatting in the table indicates statistically significant differences among the corresponding means, as determined by the statistical analysis (p < 0.05).

At 37.5°C, the eggshell temperature during the mid-incubation period (days 11–17) was generally lowest in the glucose-injected eggs, intermediate in the control eggs, and highest in the vitamin D_3_-injected eggs. However, during the late incubation (days 18–20), the eggshell temperature was lowest in the control eggs, followed by the vitamin D_3_-injected eggs, while the glucose-injected eggs exhibited the highest values.

### Effects of *in ovo* feeding and incubation temperature on the external pipping and hatching times

The results showed that the external pipping and hatching times were significantly affected by the incubation temperature (*p* < 0.05) (**Figures 1**, **2**). The external pipping time increased as the incubation temperature decreased. Eggs incubated at 37.5°C exhibited the shortest pipping duration (450–532 h), followed by those incubated at 37.0°C (455–569 h), whereas eggs incubated at 36.5°C required the longest time (458–570 h) to reach external pipping. *In ovo* feeding treatments also influenced the pipping time. The eggs injected with glucose (451–560 h) or vitamin D_3_ (456–569 h) reached external pipping earlier than the control eggs (458–570 h). A significant interaction between incubation temperature and *in ovo* feeding was observed. Eggs incubated under 37.5°C with glucose injection exhibited the shortest pipping duration, followed by 37.0°C × glucose and 36.5°C × glucose treatments. However, non-injected eggs generally required longer durations to reach pipping.

**Figure 1:**
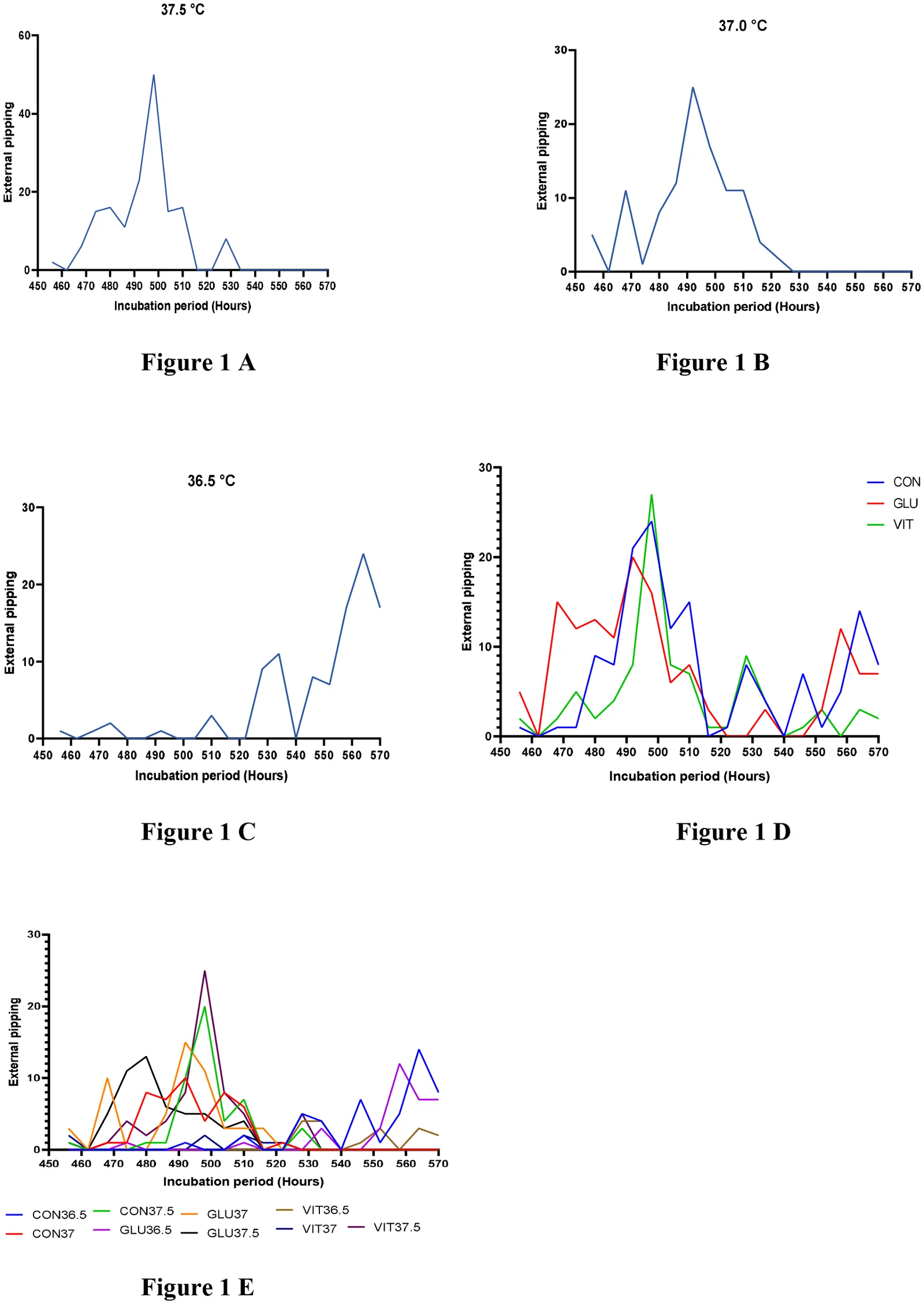
**(A–C)** Effects of incubation temperature on external pipping at 37.5°C **(A)**, 37°C **(B)**, and 36.5°C **(C)**. **(D, E)** Effects of *in ovo* feeding **(D)** and the interaction between temperature and *in ovo* feeding **(E)** on external pipping. Data are presented as the mean ± SD (*p* < 0.05).

**Figure 2:**
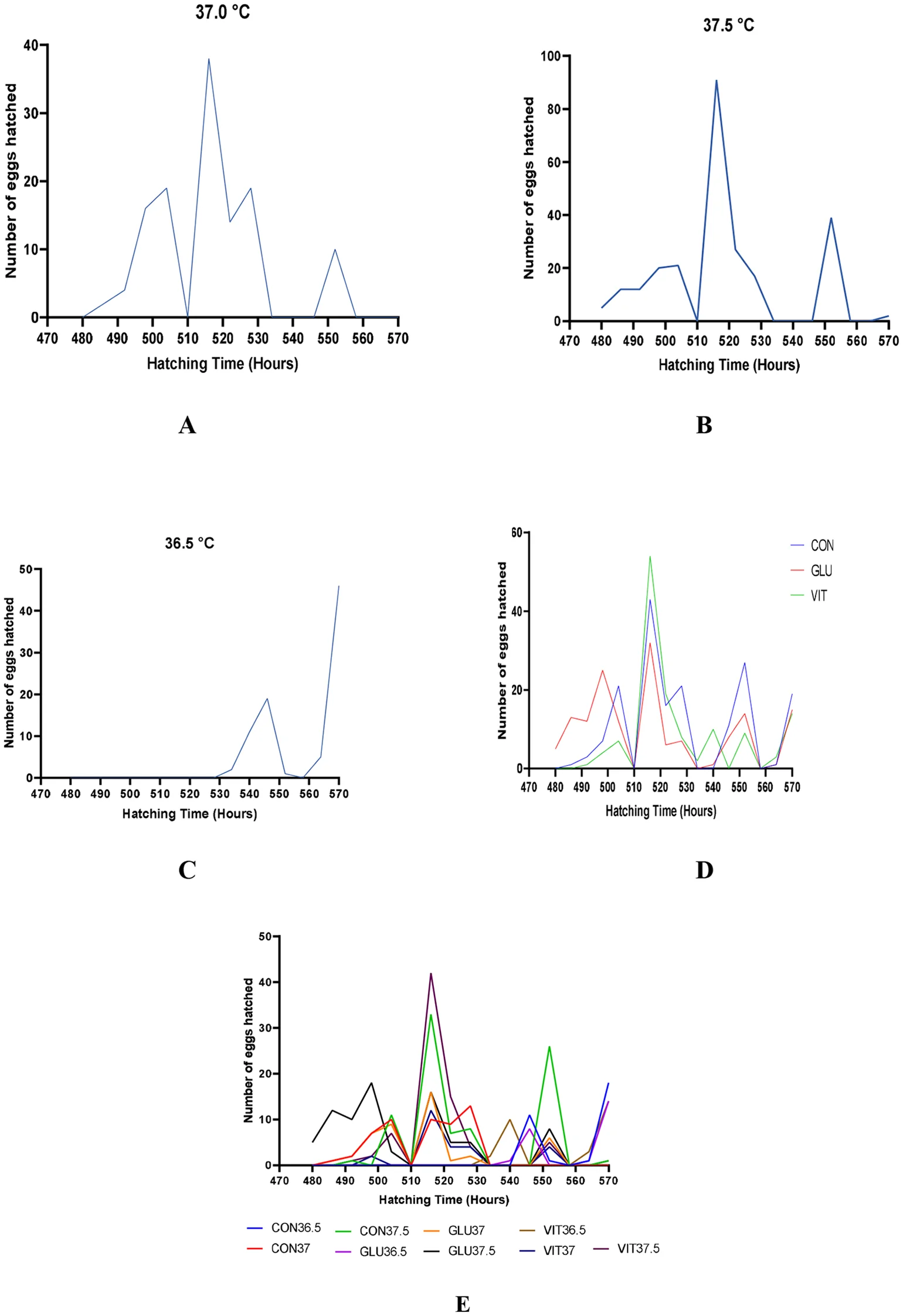
**(A–C)** Effects of incubation temperature on hatching time at 37.5°C **(A)**, 37°C **(B)**, and 36.5°C **(C)**. **(D, E)** Effects of *in ovo* feeding **(D)** and the interaction between temperature and *in ovo* feeding **(E)** on hatching time. Data are presented as the mean ± SD (*p* < 0.05).

In addition, the hatching time was also significantly influenced by the incubation temperature (*p* < 0.05). Eggs incubated at 36.5°C required the longest time to hatch (529–570 h), followed by those incubated at 37.0°C (480–569 h), whereas eggs incubated at 37.5°C hatched earlier. *In ovo* feeding affected the hatching time as well. Eggs injected with glucose hatched earlier (480–560 h) than those injected with vitamin D_3_ (456–569 h), while control eggs required the longest time to hatch (458–570 h). A significant interaction between incubation temperature and *in ovo* feeding was observed for hatching time, indicating that glucose injection combined with a higher incubation temperature shortened the duration to hatch, whereas control eggs generally exhibited longer hatching periods.

### Effects of incubation temperature and early *in ovo* feeding on embryonic mortality and hatchability

The combined effects of incubation temperature and *in ovo* feeding on hatchability are presented in **Figure 3**. A significant interaction (*p* < 0.05) was observed between incubation temperature and *in ovo* feeding treatments. Across feeding treatments, hatchability increased with increasing incubation temperature. The highest hatchability (89.86%) was recorded in glucose-injected eggs incubated at 37.5°C, whereas the lowest hatchability (48.33%) occurred in vitamin D_3_-injected eggs incubated at 36.5°C. In addition, embryonic mortality was significantly higher in the control group compared with the glucose and vitamin D_3_ groups. However, the glucose group exhibited significantly lower embryonic mortality than both the vitamin D_3_ and control groups (*p* < 0.05). With regard to the effects of temperature, the 36.5°C group recorded significantly higher embryonic mortality compared with the 37.0°C and 37.5°C groups (*p* < 0.05), whereas the 37.5°C group showed the lowest embryonic mortality among all treatment groups (*p* < 0.05) (**Figure 3**).

**Figure 3:**
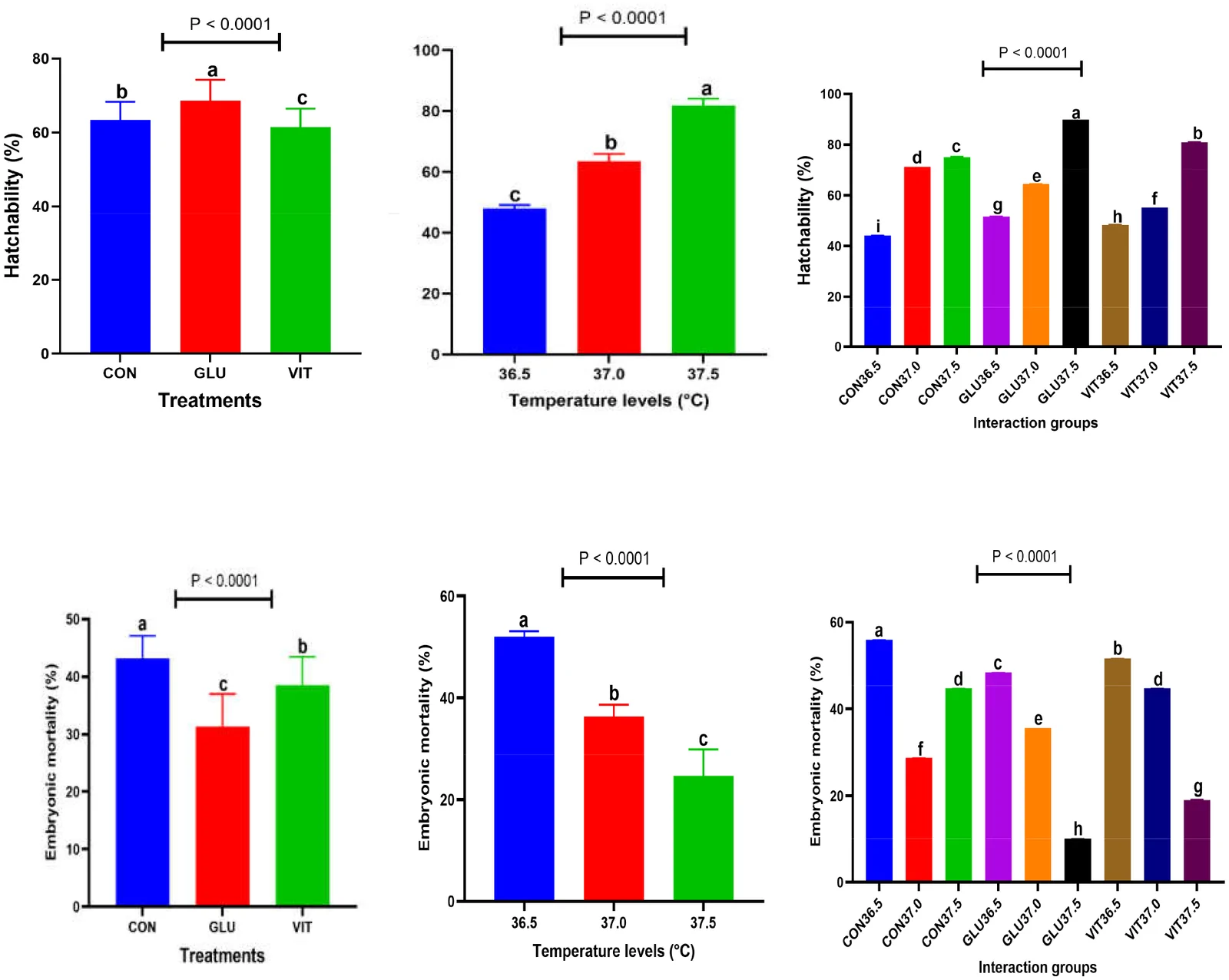
Effects of incubation temperature and early *in ovo* feeding on embryonic mortality and hatchability. Means with different lowercase letters in a column are significantly different. CON, control; GLU, glucose; VIT, vitamin; IOF, in ovo feeding; Temp, temperature; SEM, standard error of the mean. Probability value (p < 0.05).

### Effects of incubation temperature and early *in ovo* feeding on chick quality

The chick quality traits were significantly influenced by the interaction between incubation temperature and *in ovo* feeding (*p* < 0.05), as shown in **Table 3**. At 36.5°C, chicks hatched from control eggs exhibited the greatest body weight, followed by those from the vitamin D_3_-injected eggs, while the lowest body weight was observed in chicks from the glucose-injected eggs. At 37.0°C, chicks from the vitamin D_3_-injected eggs had the highest body weight, followed by those from the glucose-injected eggs and the control eggs. Chick length was highest in chicks from the control eggs (18.76 cm), followed by those from the vitamin D_3_-treated eggs (17.96 cm), and lowest in the glucose-treated eggs (17.28 cm). Shank length was highest in chicks from the glucose-injected eggs (1.84 cm), intermediate in chicks from the vitamin D_3_-treated eggs (1.72 cm), and lowest in the control group (1.70 cm).

**Table 3:** Effects of incubation temperature and early *in ovo* feeding on chick quality.

Factor	Feeding group	Chick weight (g)	Chick length (cm)	Shank length (cm)
*In ovo* feeding	CON	48.61	18.13	2.14
	GLU	46.88	17.92	1.81
	VIT	48.91	18.51	1.76
	SEM	0.992	0.183	0.134
Incubation temperature (°C)	36.5	46.31b	18.55	2.25a
	37.0	48.17ab	18.00	1.75b
	37.5	49.92a	18.01	1.71b
	SEM	0.992	0.183	0.134
*In ovo* feeding × incubation temperature (°C)	CON*36.5	47.26a	17.56bcd	1.64b
	CON*37.0	48.48a	18.76abc	1.70b
	CON*37.5	50.10a	18.08abcd	2.08a
	GLU*36.5	45.40a	19.08a	1.76b
	GLU*37.0	47.30a	17.28d	1.84b
	GLU*37.5	47.94a	17.40cd	1.82b
	VIT*36.5	46.26a	19.02a	1.72b
	VIT*37.0	48.74a	17.96abcd	1.72b
	VIT*37.5	51.72a 1.72	18.54abcd 0.317	1.84b 0.232
	SEM			
*p*-value	IOF	0.307	0.085	0.106
	Temp	0.047	0.061	0.012
	IOF*Temp	0.024	<0.0001	0.012

Means with different lowercase letters in a column are significantly different. Probability value (*p* < 0.05).

CON, control; GLU, glucose; VIT, vitamin; IOF, in ovo feeding; Temp, temperature; SEM, standard error of the mean.

### Effects of incubation temperature and early *in ovo* feeding on post-hatch performance

Post-hatch growth performance was evaluated through body weight, BWG, feed intake, and FCR at 42 days of age (**Table 4**). *In ovo* feeding significantly affected the feed intake, with glucose-treated birds showing higher feed intake than the control birds, whereas the vitamin D_3_-treated birds were intermediate. However, *in ovo* treatment did not significantly affect the body weight, BWG, or FCR. The incubation temperature significantly affected post-hatch performance. Birds originating from eggs incubated at 37.0°C had the greatest body weight (1,283.52 g), BWG (1,234.80 g), and feed intake (1,864.52 g), whereas birds from the 36.5°C treatment had the lowest values for these traits. The interaction between incubation temperature and *in ovo* treatment was significant for feed intake, with the highest value observed in the glucose × 37.0°C treatment (1,968.95 g). These findings indicate that the incubation temperature exerted a stronger influence on the overall post-hatch growth than the *in ovo* treatments, although the interaction between the two factors affected the feed intake.

**Table 4:** Effects of incubation temperature and early *in ovo* feeding on post-hatch performance.

Factor	Feeding group		Growth trait			
			Body weight (g)	Feed intake (g)	Body weight gain (g)	FCR
*In ovo* feeding		CON	1,192.14	1,570.10b	1,143.71	1.38
		GLU	1,232.65	1,803.01a	1,185.11	1.55
		VIT	1,232.66	1,697.01ab	1,184.23	1.43
		SEM	36.4	50.6	36.4	0.0742
Incubation temperature (°C)		36.5	1,104.88b	1,490.88b	1,058.58b	1.43
		37.0	1,283.52a	1,864.52a	1,234.80a	1.51
		37.5	1,269.05a	1,714.72a	1,219.67a	1.41
		SEM	36.4	50.6	36.4	0.0742
*In ovo* feeding × incubation temperature (°C)		CON*36.5	1,101.69	1,337.48c	1,054.43	1.27
		CON*37.0	1,279.65	1,671.17abc	1,229.55	1.36
		CON*37.5	1,195.09	1,701.63abc	1,147.15	1.50
		GLU*36.5	1,105.72	1,645.92abc	1,060.22	1.62
		GLU*37.0	1,291.11	1,968.95a	1,242.37	1.59
		GLU*37.5	1,301.11	1,794.15ab	1,252.63	1.44
		VIT*36.5	1,107.24	1,489.23bc	1,060.98	1.40
		VIT*37.0	1,279.79	1,953.44a	1,232.49	1.58
		VIT*37.5	1,310.94	1,648.36abc	1,259.22	1.31
		SEM	63.1	87.6	63.1	0.128
*p*-value		IOF	0.669	0.015	0.663	0.274
		Temp	0.004	<0.0001	0.005	0.638
		IOF*Temp	0.862	0.036	0.879	0.435

Means with different lowercase letters in a column are significantly different. Probability value (*p* < 0.05).

FCR, feed conversion ratio; CON, control; GLU, glucose; VIT, vitamin; IOF, in ovo feeding; Temp, temperature; SEM, standard error of the mean.

## Discussion

Eggshell temperature is a critical indicator of embryonic metabolic activity and thermal balance during incubation, and it strongly influences embryonic development, hatchability, chick quality, and subsequent growth performance (Wijnen et al., 2020; Okai et al., 2025b). Therefore, maintaining an optimal thermal environment throughout incubation is essential for successful hatchery outcomes. In the present study, the eggshell temperature prior to *in ovo* feeding showed an irregular but overall declining pattern across incubation days regardless of the incubation temperature applied. This trend likely reflects the dynamic balance between embryonic heat production and heat dissipation during early embryogenesis, when the metabolic heat production is still relatively low and embryos remain highly sensitive to environmental temperature fluctuations. Following *in ovo* feeding, a significant interaction between incubation temperature and nutrient supplementation was observed for eggshell temperature throughout the incubation period. Specifically, glucose supplementation combined with incubation at 37.5°C tended to reduce the eggshell temperature during several incubation days. Eggshell temperature typically reflects the embryo metabolic rate and the capacity of the incubator environment to dissipate metabolic heat (Uddin and Hamidu, 2014). The irregular fluctuations observed in the present study suggest that both nutrient availability and incubation temperature influenced the embryonic metabolic activity. Although *in ovo* feeding is intended to enhance the nutrient supply to the developing embryo, it may also alter metabolic processes and thereby influence eggshell temperature (Zhai et al., 2011). Previous studies reported increases in eggshell temperature following *in ovo* injection of nutrients such as vitamin D_3_ and amino acids (Fatemi et al., 2020; Molenaar et al., 2010). The gradual reduction in eggshell temperature observed during the late incubation phase (days 17–20) among *in ovo*-fed eggs may indicate a reduction in embryonic metabolic heat production as embryos approach hatching and shift physiological priorities toward pipping and hatching processes (Uni et al., 2005). Although 37.5°C is widely used as a conventional machine air incubation temperature, the present findings should not be interpreted as an indication that 36.5°C or 37.0°C is a preferable commercial incubation setting. Rather, the lower temperatures were included as experimental conditions to determine whether the response to *in ovo* supplementation changes when the thermal environment is altered. This distinction is important because machine air temperature does not directly represent embryonic temperature. As embryonic metabolism increases during incubation, metabolic heat production contributes to the eggshell temperature, making eggshell temperature a useful physiological indicator of the thermal conditions experienced by the embryo. The present study therefore combined programmed machine temperature with eggshell temperature measurements to distinguish the experimental thermal treatment from the physiological thermal response of the developing embryo. The observed temperature × *in ovo* treatment interactions further support the value of examining these factors simultaneously rather than considering 37.5°C as an isolated experimental condition.

The results obtained in this study also demonstrated a significant interaction between incubation temperature and *in ovo* feeding on hatchability. Hatchability generally increased with increasing incubation temperature, with the highest value observed in the glucose-injected eggs incubated at 37.5°C. Glucose serves as an immediate energy source for developing embryos, particularly during the late stages of incubation when the glycogen reserves may become limited (Zhang et al., 2016). The improved hatchability observed in glucose-injected eggs may therefore be associated with enhanced embryonic energy availability during critical developmental stages. Similar improvements in hatchability following *in ovo* nutrient supplementation have been reported previously (Li et al., 2016; Molenaar et al., 2010). However, the results across studies remain inconsistent. For example, Ipek et al. (2004) reported no significant effect of glucose injection on hatchability, whereas Cardinal et al. (2015) and Retes et al. (2018) observed reduced hatchability following carbohydrate supplementation. Such discrepancies may arise from differences in the incubation temperature, the timing and site of injection, the dosage of the injected nutrients, and the genetic background of the embryos used in different studies (Salmanzadeh, 2012; Alves et al., 2020). However, vitamin D_3_ injection at the lowest incubation temperature (36.5°C) resulted in a markedly reduced hatchability. This outcome may reflect a metabolic mismatch between the physiological requirements of embryonic development and the prevailing thermal environment. Vitamin D_3_ plays an essential role in calcium and phosphorus metabolism and is critical for skeletal mineralization during embryonic development (Kovacs and Ward, 2026). However, its biological activity depends on enzymatic conversion and adequate metabolic activity within the embryo. Lower incubation temperatures may reduce the embryonic metabolic rate, potentially impairing vitamin D_3_ activation and subsequent mineral metabolism. As a result, skeletal development and the overall embryonic viability may be compromised, thereby increasing embryonic mortality and reducing hatchability.

*In ovo* feeding with vitamin D_3_ improves chick quality and growth and increases hatchability because early nutrient supplementation can enhance cell proliferation, organogenesis, and nutrient uptake (Okai et al., 2026). This is consistent with the results obtained in the present study. The chick quality parameters were also significantly influenced by the interaction between incubation temperature and *in ovo* feeding. Chick weight increased progressively with increasing incubation temperature across treatments, suggesting improved embryonic growth under warmer incubation conditions within the tested range. Previous studies have similarly reported that the incubation temperature affects chick quality traits such as body weight, chick length, and structural development (Hamidu et al., 2018). Although chick length exhibited some variability across treatments, the general pattern of increased body weight at higher incubation temperatures aligns with earlier findings indicating that moderate increases in incubation temperature can enhance the embryonic growth rate (Lourens et al., 2005). Shank length, which is widely regarded as an indicator of skeletal development, displayed a temperature-dependent increase in the control and vitamin D_3_ groups, while irregular patterns were observed in glucose-fed chicks. Enhanced skeletal growth at optimal temperatures may reflect improved mineral deposition and bone formation during embryogenesis (Maatjens et al., 2014). The relatively consistent increase in shank length in the vitamin D_3_ group may be linked to the role of vitamin D_3_ in regulating calcium absorption and bone mineralization. However, the fluctuating pattern observed in the glucose-injected groups may indicate an altered nutrient partitioning between soft tissue growth and skeletal development.

The post-hatch responses further demonstrated that the effect of incubation temperature was dependent on the specific trait evaluated and, for feed intake, on the *in ovo* treatment. A significant incubation temperature × *in ovo* feeding interaction was observed for feed intake, with the highest intake observed in birds originating from glucose-injected eggs incubated at 37.0°C, followed by those from vitamin D_3_-injected eggs at the same temperature. This interaction indicates that the effect of embryonic nutrient exposure on subsequent feed consumption was not uniform across incubation temperatures. However, the interaction was not significant for body weight, BWG, or FCR, indicating that these traits should not be interpreted as evidence of a temperature-dependent supplementation response. For these traits, the incubation temperature exerted significant main effects, with birds originating from eggs incubated at 37.0°C showing greater body weight and BWG than those originating from eggs incubated at 36.5°C. These findings suggest that the incubation temperature can have persistent effects on post-hatch growth, while the additional contribution of *in ovo* supplementation may be trait-specific. Thus, the present results support the interpretation that embryonic thermal conditions and nutrient availability can interact for selected post-hatch outcomes rather than producing a uniform interaction across all growth traits. Previous studies have demonstrated that optimal incubation temperature promotes endocrine balance and metabolic efficiency, whereas suboptimal temperatures can disrupt thyroid hormone regulation and impair growth and health (Veerle et al., 2000; Yalcin et al., 2022; Tona et al., 2022). Although *in ovo* feeding has been reported to improve nutrient utilization and feed efficiency in some studies (Roto et al., 2016; Sonálio et al., 2025), the present findings suggest that its benefits may be strongly dependent on the prevailing incubation conditions. Similar improvements in post-hatch performance following *in ovo* supplementation with carbohydrates or lipids have been reported previously (Bhanja and Mandal, 2005; Yuan-Hao et al., 2024; Lu et al., 2025). Increased feed intake after hatch has also been associated with early nutrient exposure through *in ovo* feeding (Jha et al., 2019).

## Conclusion

In conclusion, the present study demonstrated that the incubation temperature and *in ovo* supplementation interact to influence selected embryonic and post-hatch responses in broiler chickens. The interaction was particularly evident for eggshell temperature, hatching characteristics, hatchability, and chick quality, while the post-hatch interaction was significant for feed intake, but not for body weight, BWG, or FCR. Glucose supplementation at 37.5°C produced the highest hatchability, whereas birds originating from eggs incubated at 37.0°C exhibited superior post-hatch body weight and BWG. These findings indicate that the biological response to *in ovo* nutrient supplementation is dependent, at least for certain traits, on the thermal environment during embryonic development. Importantly, the lower incubation temperatures evaluated in this study are experimental conditions used to investigate temperature-dependent responses and should not be interpreted as recommendations to replace conventional commercial incubation settings. Further studies involving dose–response designs, physiological mechanisms, commercial-scale application, and economic feasibility are warranted to determine the practical relevance of these interactions.

## Data Availability

The original contributions presented in the study are included in the article/supplementary material. Further inquiries can be directed to the corresponding author.
